# Prioritization of Novel Agents for Patients with Rhabdomyosarcoma: A Report from the Children’s Oncology Group (COG) New Agents for Rhabdomyosarcoma Task Force

**DOI:** 10.3390/jcm10071416

**Published:** 2021-04-01

**Authors:** Holly L. Pacenta, Wendy Allen-Rhoades, David Langenau, Peter J. Houghton, Charles Keller, Christine M. Heske, Michael D. Deel, Corinne M. Linardic, Jack F. Shern, Elizabeth Stewart, Brian Turpin, Douglas J. Harrison, Javed Khan, Leo Mascarenhas, Stephen X. Skapek, William H. Meyer, Douglas S. Hawkins, Eleanor Y. Chen, James F. Amatruda, Pooja Hingorani, Theodore W. Laetsch

**Affiliations:** 1 Cook Children’s Medical Center, Division of Hematology and Oncology, Fort Worth, TX 76014, USA; holly.pacenta@cookchildrens.org; 2 Department of Pediatrics and Harold C. Simmons Comprehensive Cancer Center, University of Texas Southwestern Medical Center, Dallas, TX 75390, USA; stephen.skapek@utsouthwestern.edu (S.X.S.); LAETSCHT@chop.edu (T.W.L.); 3 Division of Pediatric Oncology, Mayo Clinic, Rochester, MN 55902, USA; Allen-Rhoades.Wendy@mayo.edu; 4 Molecular Pathology Unit, Massachusetts General Hospital Research Institute, Charlestown, MA 02114, USA; dlangenau@mgh.harvard.edu; 5 Department of Molecular Medicine, Greehey Children’s Cancer Research Institute, San Antonio, TX 78229, USA; houghtonp@uthscsa.edu; 6 Children’s Cancer Therapy Development Institute, Beaverton, OR 97005, USA; charles@cctdi.org; 7 Pediatric Oncology Branch, Center for Cancer Research, National Cancer Institute, Bethesda, MD 20892, USA; christine.heske@nih.gov (C.M.H.); john.shern@nih.gov (J.F.S.); 8 Department of Pediatrics, Duke University School of Medicine, Durham, NC 27710, USA; michael.deel@dm.duke.edu (M.D.D.); corinne.linardic@duke.edu (C.M.L.); 9 Department of Oncology, St. Jude Children’s Research Hospital, Memphis, TN 38105, USA; elizabeth.stewart@stjude.org; 10 Division of Oncology, Cincinnati Children’s Hospital Medical Center, Cincinnati, OH 45229, USA; brian.turpin@cchmc.org; 11 Division of Pediatrics, University of Texas MD Anderson Cancer Center, Houston, TX 77030, USA; djharrison@mdanderson.org; 12 Genetics Branch, Center for Cancer Research, National Cancer Institute, Bethesda, MD 20892, USA; khanjav@mail.nih.gov; 13 Cancer and Blood Disease Institute, Children’s Hospital of Los Angeles, Los Angeles, CA 90027, USA; lmascarenhas@chla.usc.edu (L.M.); jamatruda@chla.usc.edu (J.F.A.); 14 Keck School of Medicine, University of Southern California, Los Angeles, CA 90033, USA; 15 Department of Pediatrics, Jimmy Everest Section of Pediatric Hematology/Oncology, University of Oklahoma Health Sciences Center, Oklahoma, OK 73104, USA; williammeyer@ouhsc.edu; 16 Division of Hematology/Oncology, Seattle Children’s Hospital, University of Washington, Seattle, WA 98105, USA; doug.hawkins@seattlechildrens.org; 17 Department of Laboratory Medicine and Pathology, University of Washington, Seattle, WA 98195, USA; eleanor2@uw.edu; 18 Division of Oncology and Center for Childhood Cancer Research, Children’s Hospital of Philadelphia, Philadelphia, PA 19104, USA; 19 Department of Pediatrics and Abramson Cancer Center, University of Pennsylvania, Philadelphia, PA 19104, USA

**Keywords:** rhabdomyosarcoma, clinical trials, metastasis, relapse, new agents

## Abstract

Rhabdomyosarcoma is the most common soft tissue sarcoma diagnosed in children and adolescents. Patients that are diagnosed with advanced or relapsed disease have exceptionally poor outcomes. The Children’s Oncology Group (COG) convened a rhabdomyosarcoma new agent task force in 2020 to systematically evaluate novel agents for inclusion in phase 2 or phase 3 clinical trials for patients diagnosed with rhabdomyosarcoma, following a similar effort for Ewing sarcoma. The task force was comprised of clinicians and basic scientists who collectively identified new agents for evaluation and prioritization in clinical trial testing. Here, we report the work of the task force including the framework upon which the decisions were rendered and review the top classes of agents that were discussed. Representative agents include poly-ADP-ribose polymerase (PARP) inhibitors in combination with cytotoxic agents, mitogen-activated protein kinase (MEK) inhibitors in combination with type 1 insulin-like growth factor receptor (IGFR1) inhibitors, histone deacetylase (HDAC) inhibitors, and novel cytotoxic agents.

## 1. Introduction

Rhabdomyosarcoma (RMS) is the most common soft tissue sarcoma in children and adolescents, with approximately 350 children diagnosed annually in the United States [1]. Historically, the two main subtypes of RMS were classified by histologic characteristics and designated as alveolar RMS (ARMS) and embryonal RMS (ERMS) [2,3]. More contemporary classification utilizes the presence of molecular translocations to classify RMS into fusion-positive RMS (FP-RMS) and fusion-negative RMS (FN-RMS). This designation is based on the presence of a *PAX3/7-FOXO1* fusion gene in most cases [4,5]. Approximately 80% of the cases previously categorized as ARMS harbor one of these translocations and are classified as FP-RMS, whereas ERMS and fusion negative ARMS are classified as FN-RMS [2,6,7]. Hereafter, FP-RMS or FN-RMS will be used, except when referencing previously conducted trials or studies.

The five-year overall survival of pediatric RMS is approximately 70%, although for high-risk patient groups the outcomes are poor [3,8]. Patients with stage 4 disease, other than those who are less than 10 years of age with stage 4 ERMS, have a 3-year event free survival (EFS) of less than 25% [3,9,10,11]. Recent studies for patients with metastatic RMS have attempted to intensify chemotherapy and incorporate new agents such as cixutumumab, irinotecan, and temozolomide, but those studies have not improved cure rates for this group of patients [12,13,14]. These inferior outcomes highlight the need to evaluate novel therapeutics for RMS. However, the inherent difficulties of designing and executing clinical trials to evaluate new agents in a rare disease such as RMS make it essential to conduct a comprehensive and critical evaluation of the appropriateness of testing each new agent in this population. Prior working groups for osteosarcoma and Ewing sarcoma within the Children’s Oncology Group (COG) have been useful for disease-specific drug identification, evaluation, and prioritization of agents for evaluation in clinical trials [15,16]. To this end, the COG Soft Tissue Sarcoma Committee established the New Agents for RMS Task Force.

The goal of the RMS Task Force was to bring together laboratory scientists, clinical investigators, and clinicians to identify, evaluate, and prioritize new agents for the treatment of RMS. In this report, we summarize the framework that was utilized to prioritize potential agents for clinical investigation, and we review the group’s analysis and discussion of the proposed agents.

## 2. Modified Framework for Assessing Novel Agents in Rhabdomyosarcoma

**Figure 1:**
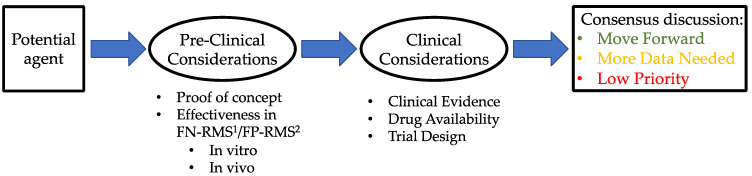
Framework used by the task force to evaluate new agents. ^1^ Fusion-positive rhabdomyosarcoma. ^2^ Fusion-negative rhabdomyosarcoma.

The RMS task force modified the framework created by the Ewing sarcoma task force to define the key criteria specific to RMS to systematically identify and evaluate new agents based on clinical and non-clinical criteria (Figure 1) [16].

### 2.1. Non-Clinical Criteria

The non-clinical criteria utilized in the modified RMS framework that remained unchanged from the Ewing sarcoma framework were: (1) evidence that the target was critical to RMS tumorigenesis or had specific expression in RMS; and (2) proof of concept data that showed the drug is active against the intended target, including in vitro and in vivo activity in RMS cell lines and animal models. In contrast to Ewing sarcoma where most tumors exhibit a characteristic fusion of EWS-FLI1, the genetic makeup of RMS is more diverse. Hence, drug candidates were considered within the context of RMS subtypes: FN-RMS, FP-RMS, as well as drugs that are active in both subtypes. The goal was to conduct a broad search to identify novel agents that would be the most useful in treating this patient population.

### 2.2. Clinical Criteria

The clinical criteria utilized in the modified RMS framework that remained unchanged from the Ewing sarcoma framework were: (1) signal of activity in early phase testing; (2) drug availability—either US Food and Drug Administration (FDA) approved or included in the Cancer Therapy Evaluation Program (CTEP) portfolio, or collaboration with an industry partner; (3) availability of a recommended pediatric phase 2 dose (RP2D); and (4) feasibility in combination therapy with currently used therapeutics for RMS.

## 3. Using the Framework to Critically Evaluate a Model Agent: Temsirolimus

Temsirolimus, a prodrug of sirolimus (rapamycin), inhibits activation of the mammalian target of rapamycin (mTOR) pathway, which has been shown to be involved in both FP-RMS and FN-RMS oncogenesis. In preclinical studies, RMS cell lines are sensitive to sirolimus and temsirolimus, and both drugs have also shown activity in RMS xenografts by both inducing tumor regression and prolonging survival [17,18,19]. Sirolimus was also shown to enhance the activity of vincristine and cyclophosphamide in RMS xenografts [20]. Temsirolimus was evaluated in phase 1 trials in both adults and children, and a pediatric RP2D was identified [21,22]. Thus, temsirolimus met the criteria for an agent of high priority with (1) evidence that the target was important to RMS oncogenesis; (2) proof of concept in vitro and in vivo activity in RMS; (3) signal of activity in RMS; (4) recommended phase 2 dosing; and (5) proof of feasibility in combination [17,18,19,20,21,22,23,24]. Temsirolimus was subsequently studied in a phase 2 trial (NCT01222715) for patients with relapsed RMS that compared bevacizumab to temsirolimus, where both agents were administered in combination with cyclophosphamide and vinorelbine. The results of this trial show that patients treated on the temsirolimus arm had a superior 6-month EFS and higher response rates compared to those treated with bevacizumab [25]. Based on this evidence, temsirolimus was moved into phase 3 testing for intermediate risk RMS patients in 2014 on ARST1431 (NCT02567435). The success of the phase 2 testing of temsirolimus provides evidence that formal evaluation using the modified framework is beneficial in systematically prioritizing agents for future testing in cooperative group trials.

## 4. Using the Model to Evaluate Novel Agents for Use in Future Clinical Trials

Agents were nominated by committee members for evaluation and discussion by the group. The full list of the nominated agents is listed in Table 1 and the top five agents based on the task force rankings are designated in bold font. The data available for each agent were presented to the task force and evaluated using the above criteria. After all the potential agents were presented, task force members were asked to submit a prioritization list. Below, we review the available data and the group’s discussion of the top five classes of agents based on the task force ranking, which are summarized in Table 2.

## 5. Poly-ADP-Ribose Polymerase Inhibitors in Combination with Cytotoxic Agents

Poly-ADP-ribose polymerases (PARP) are nuclear enzymes that are involved in repairing DNA damage. These enzymes (PARP1, PARP2, and PARP3) are overexpressed in both FP-RMS and FN-RMS tumor samples [26]. PARP1 is the primary enzyme of the PARP family and binds to DNA single-strand breaks to facilitate repair [27]. The inhibition of PARP leads to the accumulation of DNA strand breaks and subsequently apoptosis and cell death [27]. Olaparib, an inhibitor of PARP1 and PARP2, also leads to trapping of the PARP enzyme on damaged DNA and may result in improved cytotoxicity [28]. Due to efficacy in preclinical models and early phase studies in adult malignancies, PARP inhibition is a therapeutic target of interest in RMS that was ranked highly by the task force.

PARP inhibition was evaluated in vitro in FN-RMS and FP-RMS cell lines using multiple agents in this class. Olaparib (inhibitor of PARP 1/2) and AZD2461 (inhibitor of PARP 1/2/3) resulted in a reduction in growth at clinically achievable concentrations (1–5 μM olaparib and 5–10 μM AZD2461); however, talazoparib (inhibitor of PARP 1/2) was not effective against FP-RMS or FN-RMS [26,29,30]. Preclinical combination studies in RMS have shown that PARP inhibitors can enhance the efficacy of topoisomerase II inhibitors, ionizing radiation, and alkylating agents such as temozolomide [26,29,31,32]. The combination of olaparib with temozolomide was particularly effective when tested in mouse and zebrafish xenografts [33]. This combination led to a potent reduction in tumor size in both FN-RMS and FP-RMS in each of the tested models, whereas treatment with each drug individually had limited responses [33].

In the clinical setting, multiple PARP inhibitors, including olaparib, rucaparib, niraparib, and talazoparib, have been FDA-approved as single agents for use in adult cancers. PARP inhibitors have also been studied in clinical trials in combination with other cytotoxic agents, including temozolomide, topotecan, paclitaxel, and cyclophosphamide [34,35,36,37,38,39,40]. However, the combination of PARP inhibitors with cytotoxic chemotherapy led to increased toxicity, particularly myelosuppression, that required dose reductions in several studies [40,41,42,43]. PARP inhibition in RMS is currently being studied in an ongoing phase 1 clinical trial evaluating olaparib in combination with temozolomide in Ewing sarcoma and RMS in patients aged 16 years and older (NCT01858168).

The combination of a PARP inhibitor with cytotoxic agents, specifically temozolomide, was rated highly by the group due to their availability, as both agents are FDA-approved. Prior studies suggest that dose reductions are indicated given the increased risk of myelosuppression with the combination, and data from the ongoing phase 1 study in RMS is not yet available. In preclinical studies, the combination was effective in FN-RMS and FP-RMS xenograft models, but the group determined that additional preclinical studies evaluating PARP inhibitors in combination with other less myelotoxic agents, such as irinotecan, would be beneficial. Given the ongoing trial of the combination in adolescents and adults with RMS, the committee preferred to await the results of this study before proceeding with a new clinical trial for children with RMS utilizing this combination.

## 6. MEK Inhibitors in Combination with Type 1 Insulin-Like Growth Factor Receptor Inhibitors

Another combination of interest was the inhibition of mitogen-activated protein kinase (MEK) and type 1 insulin-like growth factor receptor (IGF1R). RAS pathway mutations are reported in approximately 50% of FN-RMS which leads to the activation of MEK1/2 [44,45]. Preclinical studies have demonstrated that RAS is an oncogenic driver in FN-RMS, suggesting that targeting this pathway may be effective treatment [46,47,48,49,50]. IGF1R is a receptor tyrosine kinase of interest in the treatment of RMS because it is overexpressed in both FN-RMS and FP-RMS and IGF1R inhibition was effective in preclinical studies of FN-RMS and FP-RMS [51,52,53,54,55].

In preclinical studies of RAS-mutated FN-RMS, treatment with trametinib, a selective inhibitor of MEK1/2, led to decreased cell viability when tested in vitro and also resulted in decreased tumor volume and prolonged survival in mouse xenografts [56]. However, the effect of trametinib was limited in xenograft models, with animals ultimately developing disease progression [56]. When trametinib was administered in combination with BMS-754807, a small molecule inhibitor of IGF1R and the insulin receptor, there was a more profound decrease in cell viability and delay in tumor growth [56].

Based on the promising preclinical data of the combination of MEK and IGF1R inhibition in RAS mutated FN-RMS, the task force discussed the possibility of evaluating this combination in a clinical trial for this patient population. There are four FDA-approved inhibitors of MEK1/2, including trametinib, binimetinib, selumetinib, and cobimetinib [57,58,59,60]. Selumetinib was recently approved for children with neurofibromatosis type 1 who have inoperable plexiform neurofibromas. Selumetinib was well tolerated in the clinical trials leading to its FDA approval in April 2020 for neurofibromatosis type 1 and has also demonstrated activity in children with low grade gliomas [61,62,63,64]. Trametinib has also been studied in pediatrics and was well tolerated in a phase 1 study in patients with a low-grade glioma or plexiform neurofibroma and is now being studied in an ongoing phase 2 study (NCT03363217) [65].

The main categories of IGF1R inhibitors include monoclonal antibodies that target IGF1R or its ligands (IGF-1 and IGF-2), or IGF1R tyrosine kinase inhibitors [66,67]. While there are several FDA-approved MEK inhibitors, there are no FDA-approved IGF1R inhibitors. Overall the efficacy of IGF1R inhibition in clinical trials has been limited, and the current focus is to identify new combinations of IGF1R inhibitors with novel agents that may lead to improved efficacy, as well as identify biomarkers to predict patient subgroups which will respond to IGF1R inhibition [66,67,68].

A number of clinical trials of IGF1R inhibitors for patients with RMS have been conducted, including a phase 2 study where R1507, a monoclonal antibody targeting IGF1, was studied in adults with relapsed or refractory sarcomas [69]. There were 36 individuals with RMS enrolled in this study (ARMS: 12, ERMS: 3, unknown type: 21) and one patient with ERMS experienced a confirmed partial response, while three patients with RMS experienced short-lived decreases in tumor size of greater than 50% [69]. In pediatric RMS, a recent COG trial evaluated the addition of cixutumumab (monoclonal antibody against IGF1R) or temozolomide to cytotoxic chemotherapy in unselected patients with metastatic RMS, and found that neither agent improved outcomes [12]. An ongoing phase 2 clinical trial based on preclinical data evaluating the combination of ganitumab (monoclonal antibody targeting IGF1R) with dasatinib (a multi-kinase inhibitor) in children and adults with RMS (NCT03041701) aims to determine whether this combination can produce more durable responses [70].

IGF1R inhibitors have not been evaluated in combination with MEK inhibitors in clinical trials for patients with RMS. However, the combination of selumetinib with cixutumumab was recently evaluated in adults with advanced malignancies, and an early report from the study noted one dose limiting toxicity of visual changes, and one partial response [71]. Based on the preclinical data of MEK inhibition and IGF1R inhibition in RAS mutated FN-RMS, this may be a subset of patients who will respond to this combination of agents. Although this combination was of interest to the task force, it was determined that additional preclinical data were needed to determine whether the exposures achievable in humans are effective in preclinical studies.

## 7. Histone Deacetylase Inhibitors

Epigenetic processes control gene expression through histone acetylation and deacetylation, and are important in oncogenesis. In certain cancers, there is dysregulation of these histone acetylation processes [72]. Histone deacetylase (HDAC) inhibitors are a class of medications that can reactivate proapoptotic genes that have been suppressed in tumor cells. Treatment with HDAC inhibitors in preclinical studies of FP-RMS and FN-RMS led to reduced cell growth, apoptosis, and induced differentiation of RMS cells [73,74,75]. HDAC inhibition is of interest particularly in FP-RMS because the fusion protein PAX3/7-FOXO1 is an oncogenic driver that is epigenetically regulated [76,77]. Specifically, an in vitro study demonstrated that treatment with entinostat, a selective class I and class IV HDAC inhibitor, led to HDAC inhibition and suppressed the expression of PAX3-FOXO1 and PAX7-FOXO1 or its downstream effects in FP-RMS cells [77,78,79].

The activity of entinostat in RMS was evaluated in two xenograft studies, and the researchers drew different conclusions. Keller et al. studied the effectiveness of entinostat in PDX models of FN-RMS and FP-RMS. In these studies, entinostat was administered daily at dose of 4 mg/kg for 21 days [78,80]. This dosing schedule led to decreased growth of FN-RMS and FP-RMS tumors in mice, and in FP-RMS PDX models the combination of entinostat with vincristine was more effective than either agent alone [78]. Preclinical work also demonstrated that HDAC inhibition leads to the disruption of the core regulatory circuitry of FP-RMS, and treatment with entinostat in FP-RMS led to a decrease in PAX3-FOXO1 protein levels via inhibition of HDAC3 [77,78,79]. However, in the experiments performed by Houghton et al. as part of the National Cancer Institute (NCI)-sponsored Pediatric Preclinical Testing Program, entinostat was administered twice daily at a dose of 2.5 mg/kg for 4 days over 3 consecutive weeks in xenograft models of both FN-RMS and FP-RMS. In this study, the effect of entinostat on tumor growth was not significant in the majority of FP-RMS xenograft models, and no effect was observed in mice bearing FN-RMS tumors [81]. Additionally, there was no increased activity when entinostat was administered in combination with other cytotoxic chemotherapy agents [81].

The results from these conflicting studies were discussed in detail by the task force, but it is difficult to compare the results, as different dosing strategies were used for the xenograft experiments. In humans, entinostat has a half-life of 50 h and is administered weekly, but in mice the half-life is much shorter and requires more frequent dosing [72,78,81]. The pharmacokinetic (PK) data from both studies were analyzed by two independent pharmacologists to assist in the data comparison given the different dosing schedules. However, it was concluded that the PK data in mice were difficult to extrapolate to humans. Mice can tolerate higher dose levels without toxicity compared to humans, and when tested in humans, it is possible that the maximum tolerated dose may not reach the point of clinical efficacy.

There are multiple FDA-approved HDAC inhibitors for hematologic malignancies in adults including vorinostat, belinostat, and romidepsin, which are approved for T-cell lymphoma; and panobinostat which is approved for multiple myeloma [82]. Entinostat is not FDA-approved, but has been evaluated in clinical trials for patients with breast cancer [83]. HDAC inhibitors have been evaluated in clinical trials for adults with sarcomas and have demonstrated limited success as monotherapy [82]. In pediatrics, HDAC inhibitors have been evaluated as single agents in phase 1 clinical trials which included patients with RMS and while these agents were well tolerated, there were no objective responses reported [84,85,86,87,88]. These agents may be more effective in combination with chemotherapy or other targeted agents, and there is an ongoing clinical trial for individuals with RMS aged 16 years and older studying the HDAC inhibitor mocetinostat in combination with vinorelbine (NCT04299113).

Based on the difficulties extrapolating the mouse xenograft responses and PK data to humans, along with the conflicting results, and limited efficacy of HDAC inhibitors in pediatric phase 1 trials, the task force determined that more preclinical testing is needed to further investigate the effectiveness of entinostat in RMS before proceeding with a new prospective clinical trial for patients with RMS.

## 8. Novel Cytotoxic Agents: PLX038

PLX038 is earlier in development compared to many of the other agents discussed, but was highly rated amongst the group. PLX038 is a pegylated prodrug of anticancer agent SN-38, which is the active metabolite of irinotecan. Irinotecan is a topoisomerase I inhibitor that inhibits the repair of single strand DNA breaks and has demonstrated activity in RMS [13,89,90]. In comparison to irinotecan, PLX038 leads to the sustained release of SN-38, which may result in improved efficacy over conventional topoisomerase I inhibitors due to the accumulation of the drug within tumors [91]. This agent was evaluated in 32 xenograft models of pediatric cancers, including RMS [92]. PLX038 was highly active, with 78% of the xenografts experiencing more than a 50% reduction in tumor size after one dose [92]. Additionally, PLX038 showed equal or slightly improved responses in the same study when compared to irinotecan. In the clinical setting, PLX038 is under investigation in two ongoing clinical trials. The first is a phase 1 study of single agent PLX038 in adults with solid tumors (NCT04209595). The second is a phase 1 study at the NCI evaluating the combination of PLX038 and a PARP inhibitor rucaparib (NCT04209595), which recently enrolled the first patient [93].

PLX038 warrants further preclinical study in RMS and in combination with other agents such as vincristine and temozolomide, as these have demonstrated improved outcomes with limited overlapping toxicities in pediatric sarcomas when used in combination with irinotecan. Additionally, the results from the ongoing phase 1 studies in adults will be beneficial in identifying a dose for use in a future pediatric study.

## 9. Novel Cytotoxic Agents: Eribulin

Microtubule inhibitors such as vincristine are a mainstay in the treatment of RMS, but can cause peripheral neuropathy, which limits their use [94]. Other microtubule inhibitors include taxanes, which are microtubule stabilizing agents, but these agents have had limited success in pediatric malignancies [95,96]. Eribulin is a novel microtubule inhibitor which is a synthetic analogue of the natural product halichondrin B, derived from the marine sponge *Halichondria okadai* [97,98]. The mechanism of eribulin is unique in that it leads to apoptosis by inhibiting the polymerization of tubulin subunits, but it does not affect microtubule shortening [99].

Based on the widespread use of microtubule inhibitors in pediatrics, eribulin was studied in vitro and in vivo in the Pediatric Preclinical Testing Program [100]. Eribulin demonstrated activity in RMS and 6/7 RMS xenografts (5 FP-RMS and 1 FN-RMS) achieved a complete remission (CR) or maintained a CR in response to treatment [100]. Furthermore, there was increased activity of eribulin in the RMS xenograft models compared to vincristine. The combination of eribulin with irinotecan was synergistic in several FP-RMS xenografts, and more efficacious than the combination of vincristine and irinotecan in models with wild type TP53 [101].

In the clinical setting, eribulin is FDA-approved for adults with breast cancer and liposarcoma. In pediatric patients eribulin was evaluated in a phase 1 study of 23 pediatric patients with relapsed or refractory solid tumors [102]. While there were no patients with RMS enrolled on this study, there was one patient with Ewing sarcoma who experienced a partial response and three patients had stable disease [102]. The RP2D of eribulin was determined to be 1.4 mg/m^2^, which is the same as the adult dose [102]. There is an ongoing phase 2 study evaluating eribulin which includes pediatric patients with relapsed or refractory RMS (NCT03441360). Eribulin was also studied in combination with irinotecan in a phase 1/2 trial of children with relapsed or refractory solid tumors [103]. The results from the phase 1 portion of the study were recently reported [103]. There were 13 patients enrolled including four individuals with RMS. There were no dose limiting toxicities reported and at the time of data cut off there were four patients who continued to receive treatment with irinotecan and eribulin, including one individual with RMS [103]. The phase 2 portion of the study is ongoing (NCT03245450).

Eribulin is an agent of interest in RMS as it was effective in preclinical studies and there is an identified RP2D in pediatrics. However, microtubule inhibitors such as eribulin may overperform in mouse models as compared to humans. This may be due to their steep dose response curve suggesting that activity may drop significantly below the threshold concentration/exposure. This was seen in osteosarcoma whereby significant responses were seen in PDX models but none were observed in the phase 2 trial in patients with recurrent osteosarcoma [100,104,105]. Ultimately the recommendation of the task force was to await the results from the ongoing trials prior to designing a new clinical trial for RMS.

## 10. Other Targeted Agents

In addition to the top-rated agents reviewed above, other potential targets/agents of interest with promising early pre-clinical data are emerging. We discuss three such agents: fibroblast growth receptor 4 (FGFR4) targeted chimeric antigen receptor (CAR) T-cells, bromodomain and extraterminal domain (BET) protein inhibitors, and ephrin receptor inhibitors. While these agents are early in development, preclinical data for new promising agents are constantly being generated, and a brief summary of the available data for the three agents of interest is described below.

FGFR4 is a receptor tyrosine kinase that is expressed in FN-RMS and FP-RMS [44,106,107,108]. FGFR4 inhibition was effective in preclinical studies of FN-RMS and FP-RMS and FGFR4 is differentially expressed in RMS compared to mature skeletal muscle, therefore it may be a beneficial target for immunotherapy [109,110]. FGFR4 targeted chimeric antigen receptor (CAR) T-cells are currently in pre-clinical development and were effective against RMS in both in vitro and in vivo studies [111,112,113].

BET protein inhibitors are another promising class of agents for the treatment of FP-RMS. BET proteins are highly expressed in FP-RMS and the BET protein BRD4 is required for the PAX3-FOXO1 fusion protein to exhibit its oncogenic effects [114,115]. Preclinical studies demonstrated that BET inhibition disrupts the interaction between BRD4 and PAX3-FOXO1 leading to a reduced half-life of the fusion oncogene, and FP-RMS cell lines were susceptible to BET inhibition [114,115]. BET inhibitors also demonstrated antiangiogenic effects in FP-RMS xenograft models [116]. In adult clinical trials BET inhibitors have had limited efficacy and are not yet FDA-approved [117]. There is an ongoing trial evaluating the BET inhibitor BMS-986158 in children but results are not yet available (NCT03936465).

Erythropoietin-producing hepatocellular (Eph) proteins are a family of receptor tyrosine kinases that include Eph-A and Eph-B receptors that bind the ligands ephrin-A and ephrin-B, respectively [118]. Eph-A/ephrin-A signaling is important in myogenic differentiation and upregulation of Eph/ephrin proteins was identified in both FN and FP-RMS [119,120,121]. A preclinical study found that GLPG1790, a pan-Eph inhibitor, led to growth inhibition and promoted myogenic differentiation in FN-RMS when evaluated in vitro [120]. In contrast, inhibition of EphB4 signaling was not effective in FN-RMS, but demonstrated a decrease in tumor progression in a murine model of FP-RMS, suggesting that inhibition of EphB4 may be more effective when evaluated in combination with other agents [122]. Inhibitors of Eph/ephrin have been studied in clinical trials in adults with solid tumors, but these agents have not been studied in patients with RMS [118,123,124].

## 11. Discussion

There is a critical need for novel therapies in the treatment of RMS, particularly in patients with metastatic disease, as their outcomes remain poor. Disease-specific working groups within the COG have been useful in facilitating discussions to analyze and prioritize drugs using a multidisciplinary team of basic scientists, clinical investigators, and clinicians. Our group utilized a modified framework to identify agents for consideration in the next COG clinical trial for patients with RMS. Unique to RMS was the consideration of agents in the context of disease subtypes. Oncogenic drivers and molecular aberrations are known to differ between FP-RMS and FN-RMS, and this is important to consider when evaluating the potential efficacy of targeted therapies for RMS. Novel treatments for RMS may require investigation within a clinical trial that is subtype-specific in order to advance outcomes in this disease.

Of the agents that were considered, the top candidates included novel cytotoxic agents and targeted therapies. The overarching conclusion of the RMS new agent task force was that despite a rigorous review of several promising agents, none of the agents are currently ready for clinical trial testing by COG as more data are needed prior to evaluation in a large phase 2 or 3 clinical trial for patients with RMS. While microtubule inhibitors such as eribulin and the combination of a PARP inhibitor and temozolomide both met the criteria of the proposed framework, the group’s recommendation was to await the data from ongoing clinical trials with these agents prior to proceeding with a COG clinical trial incorporating either of these agents. Other agents had preclinical data in RMS but lacked an identified RP2D in pediatrics, such as PLX038. It was also determined that additional preclinical data were needed for HDAC inhibitors such as entinostat, and MEK inhibitors such as trametinib, particularly in terms of pharmacokinetic studies to determine whether the doses needed to attain preclinical activity will be clinically achievable.

The incorporation of new agents into RMS treatment has the potential to modify the current treatment landscape to provide improved outcomes for these patients. Novel cytotoxic agents such as eribulin and PLX038 may provide enhanced activity and ultimately replace the use of the similar cytotoxic agents vincristine and irinotecan that are an established part of chemotherapy regimens for RMS. On the other hand, targeted therapies may be most beneficial when administered with chemotherapy or in combination with other targeted agents. Suggestions for incorporating these agents in future clinical trials for RMS include the addition of targeted therapies to cytotoxic chemotherapy in patients with metastatic disease in order to improve cure rates. In patients with less advanced disease, novel agents may be evaluated either in addition to, or in place of standard chemotherapy agents in order to both improve outcomes and/or decrease toxicity. Agents which are earlier in clinical development should continue to undergo initial evaluation in studies for relapsed patients.

Although the task force concluded that additional data were needed before proceeding with a clinical trial, this review process was a useful way for experts in the field to convene and thoroughly evaluate the available preclinical and clinical data for each of the potential agents. Through these discussions, we were able to prioritize agents and identify areas where additional preclinical or early phase clinical studies were needed, or where results from ongoing trials would inform the design and/or dosing of a pediatric RMS trial. We believe that these ongoing discussions will lead to subsequent collaborative efforts between basic science researchers and clinical investigators so that deficiencies in the available data can be further investigated. With continued poor outcomes in advanced stage RMS, as well as the overall rarity of this disease, it is imperative to acquire the necessary background data before moving forward with an RMS-specific clinical trial or phase 2/3 studies.

## Figures and Tables

**Table 1:** List of agents nominated by the task force.

Class	Example Agents
Novel cytotoxic agents	**Microtubule inhibitors**: **Eribulin**
	**Topoisomerase I inhibitors**: **PLX038**, PEN-866
DNA damage/repair	**PARP ^1^ inhibitors/Cytotoxic agents**: **Olaparib/temozolomide**
Epigenetic targets	**HDAC ^2^ inhibitors**: **Entinostat**
	Bromodomain inhibitors
Immune Targets	B7-H3 inhibitors
	PD1 ^3^/PD-L1 ^4^ inhibitors
	FGFR4 ^5^ CAR T-cell ^6^
Tyrosine kinase inhibitors	Multi-targeted tyrosine kinase inhibitors: cabozantinib, regorafenib, pazopanib
	ALK ^7^ inhibitors: crizotinib
	Eph ^8^/ephrin receptor inhibitors
	**MEK ^9^ inhibitors/IGF1R ^10^ inhibitors**: **trametinib/ganitumab**
Cell cycle inhibitors	CDK4/6 ^11^ inhibitor: palbociclib
	Wee1 inhibitor
	CHK1 ^12^ inhibitor

^1^ Poly ADP-ribose polymerase. ^2^ Histone deacetylase. ^3^ Programmed cell death protein 1. ^4^ Programmed death-ligand 1. ^5^ Fibroblast growth factor receptor 4. ^6^ Chimeric antigen receptor T-cell. ^7^ Anaplastic lymphoma kinase. ^8^ Erythropoietin-producing hepatocellular. ^9^ Mitogen-activated protein kinase. ^10^ Insulin-like growth factor 1 receptor. ^11^ Cyclin-dependent kinase 4/6. ^12^ Checkpoint kinase 1.

**Table 2:** Summary of the available data for the top five agents identified by the task force.

Agent Rank	Agent	Preclinical Evidence	Adult Clinical Data	Pediatric Clinical Data	Drug Availability	Consensus Decision
In trial	temsirolimus	×	×	×	×	In trial
1	PARP ^1^ inhibitor/cytotoxic agent	×	×	Ongoing phase 1 of olaparib in combination with temozolomide for EWS ^2^ and RMS ^3^ (age ≥ 16 years)	×	Need more preclinical combination studies, need phase 1 combination data
2	MEK ^4^ inhibitor/IGF1R ^5^ inhibitor	×	×	×		Need more preclinical testing to determine if doses in in vitro studies are achievable in humans
3	PLX038	×	×			Need preclinical combination studies, need phase 1 pediatric dose
4	HDAC ^6^ inhibitor	×	×	×	×	Need more preclinical testing in vivo to mimic human PK ^7^ data
5	eribulin	×	×	Ongoing phase 2 in RMS ^3^, ongoing phase 1/2 in combination with irinotecan in R/R ^8^ solid tumors	×	Need phase 2 pediatric data

The top five agents are listed in order of their rank based on votes by the task force members. Areas where the task force felt there was sufficient evidence or data are noted with “×” and areas where there were no available data or insufficient evidence are blank. ^1^ Poly ADP-ribose polymerase. ^2^ Ewing sarcoma. ^3^ Rhabdomyosarcoma. ^4^ Mitogen-activated protein kinase. ^5^ Insulin-like growth factor 1 receptor. ^6^ Histone deacetylase. ^7^ pharmacokinetic. ^8^ Relapsed/refractory.

## Data Availability

Not applicable.

## References

[1] Howlader N., Noone A.M., Krapcho M., Miller D., Bishop K., Kosary C.L., Yu M., Ruhl J., Tatalovich Z., Mariotto A. (2016). SEER Cancer Statistics Review, 1975–2014.

[2] Parham D.M., Barr F.G. (2013). Classification of rhabdomyosarcoma and its molecular basis. Adv. Anat. Pathol..

[3] Skapek S.X., Ferrari A., Gupta A.A., Lupo P.J., Butler E., Shipley J., Barr F.G., Hawkins D.S. (2019). Rhabdomyosarcoma. Nat. Rev. Dis. Primers.

[4] Barr F.G., Galili N., Holick J., Biegel J.A., Rovera G., Emanuel B.S. (1993). Rearrangement of the PAX3 paired box gene in the paediatric solid tumour alveolar rhabdomyosarcoma. Nat. Genet..

[5] Davis R.J., D’Cruz C.M., Lovell M.A., Biegel J.A., Barr F.G. (1994). Fusion of PAX7 to FKHR by the variant t(1;13)(p36;q14) translocation in alveolar rhabdomyosarcoma. Cancer Res..

[6] Parham D.M., Qualman S.J., Teot L., Barr F.G., Morotti R., Sorensen P.H., Triche T.J., Meyer W.H. (2007). Correlation between histology and PAX/FKHR fusion status in alveolar rhabdomyosarcoma: A report from the Children’s Oncology Group. Am. J. Surg. Pathol..

[7] Rudzinski E.R., Teot L.A., Anderson J.R., Moore J., Bridge J.A., Barr F.G., Gastier-Foster J.M., Skapek S.X., Hawkins D.S., Parham D.M. (2013). Dense pattern of embryonal rhabdomyosarcoma, a lesion easily confused with alveolar rhabdomyosarcoma: A report from the Soft Tissue Sarcoma Committee of the Children’s Oncology Group. Am. J. Clin. Pathol..

[8] Chen C., Dorado Garcia H., Scheer M., Henssen A.G. (2019). Current and Future Treatment Strategies for Rhabdomyosarcoma. Front. Oncol..

[9] Malempati S., Hawkins D.S. (2012). Rhabdomyosarcoma: Review of the Children’s Oncology Group (COG) Soft-Tissue Sarcoma Committee experience and rationale for current COG studies. Pediatric Blood Cancer.

[10] Oberlin O., Rey A., Lyden E., Bisogno G., Stevens M.C., Meyer W.H., Carli M., Anderson J.R. (2008). Prognostic factors in metastatic rhabdomyosarcomas: Results of a pooled analysis from United States and European cooperative groups. J. Clin. Oncol. Off. J. Am. Soc. Clin. Oncol..

[11] Rudzinski E.R., Anderson J.R., Chi Y.Y., Gastier-Foster J.M., Astbury C., Barr F.G., Skapek S.X., Hawkins D.S., Weigel B.J., Pappo A. (2017). Histology, fusion status, and outcome in metastatic rhabdomyosarcoma: A report from the Children’s Oncology Group. Pediatr. Blood Cancer.

[12] Malempati S., Weigel B.J., Chi Y.Y., Tian J., Anderson J.R., Parham D.M., Teot L.A., Rodeberg D.A., Yock T.I., Shulkin B.L. (2019). The addition of cixutumumab or temozolomide to intensive multiagent chemotherapy is feasible but does not improve outcome for patients with metastatic rhabdomyosarcoma: A report from the Children’s Oncology Group. Cancer.

[13] Pappo A.S., Lyden E., Breitfeld P., Donaldson S.S., Wiener E., Parham D., Crews K.R., Houghton P., Meyer W.H. (2007). Two Consecutive Phase II Window Trials of Irinotecan Alone or in Combination With Vincristine for the Treatment of Metastatic Rhabdomyosarcoma: The Children’s Oncology Group. J. Clin. Oncol..

[14] Weigel B.J., Lyden E., Anderson J.R., Meyer W.H., Parham D.M., Rodeberg D.A., Michalski J.M., Hawkins D.S., Arndt C.A. (2016). Intensive Multiagent Therapy, Including Dose-Compressed Cycles of Ifosfamide/Etoposide and Vincristine/Doxorubicin/Cyclophosphamide, Irinotecan, and Radiation, in Patients With High-Risk Rhabdomyosarcoma: A Report From the Children’s Oncology Group. J. Clin. Oncol. Off. J. Am. Soc. Clin. Oncol..

[15] Khanna C., Fan T.M., Gorlick R., Helman L.J., Kleinerman E.S., Adamson P.C., Houghton P.J., Tap W.D., Welch D.R., Steeg P.S. (2014). Toward a drug development path that targets metastatic progression in osteosarcoma. Clin. Cancer Res. Off. J. Am. Assoc. Cancer Res..

[16] Bailey K., Cost C., Davis I., Glade-Bender J., Grohar P., Houghton P., Isakoff M., Stewart E., Laack N., Yustein J. (2019). Emerging novel agents for patients with advanced Ewing sarcoma: A report from the Children’s Oncology Group (COG) New Agents for Ewing Sarcoma Task Force. F1000Research.

[17] Dilling M.B., Dias P., Shapiro D.N., Germain G.S., Johnson R.K., Houghton P.J. (1994). Rapamycin selectively inhibits the growth of childhood rhabdomyosarcoma cells through inhibition of signaling via the type I insulin-like growth factor receptor. Cancer Res..

[18] Houghton P.J., Morton C.L., Kolb E.A., Gorlick R., Lock R., Carol H., Reynolds C.P., Maris J.M., Keir S.T., Billups C.A. (2008). Initial testing (stage 1) of the mTOR inhibitor rapamycin by the pediatric preclinical testing program. Pediatr. Blood Cancer.

[19] Wan X., Shen N., Mendoza A., Khanna C., Helman L.J. (2006). CCI-779 inhibits rhabdomyosarcoma xenograft growth by an antiangiogenic mechanism linked to the targeting of mTOR/Hif-1alpha/VEGF signaling. Neoplasia.

[20] Houghton P.J., Morton C.L., Gorlick R., Lock R.B., Carol H., Reynolds C.P., Kang M.H., Maris J.M., Keir S.T., Kolb E.A. (2010). Stage 2 combination testing of rapamycin with cytotoxic agents by the Pediatric Preclinical Testing Program. Mol. Cancer Ther..

[21] Raymond E., Alexandre J., Faivre S., Vera K., Materman E., Boni J., Leister C., Korth-Bradley J., Hanauske A., Armand J.P. (2004). Safety and pharmacokinetics of escalated doses of weekly intravenous infusion of CCI-779, a novel mTOR inhibitor, in patients with cancer. J. Clin. Oncol. Off. J. Am. Soc. Clin. Oncol..

[22] Spunt S.L., Grupp S.A., Vik T.A., Santana V.M., Greenblatt D.J., Clancy J., Berkenblit A., Krygowski M., Ananthakrishnan R., Boni J.P. (2011). Phase I study of temsirolimus in pediatric patients with recurrent/refractory solid tumors. J. Clin. Oncol. Off. J. Am. Soc. Clin. Oncol..

[23] Bagatell R., Norris R., Ingle A.M., Ahern C., Voss S., Fox E., Little A.R., Weigel B.J., Adamson P.C., Blaney S. (2014). Phase 1 trial of temsirolimus in combination with irinotecan and temozolomide in children, adolescents and young adults with relapsed or refractory solid tumors: A Children’s Oncology Group Study. Pediatr. Blood Cancer.

[24] Mascarenhas L., Meyer W.H., Lyden E., Rodeberg D.A., Indelicato D.J., Linardic C.M., Anderson J.R., Hawkins D.S. (2014). Randomized phase II trial of bevacizumab and temsirolimus in combination with vinorelbine (V) and cyclophosphamide (C) for first relapse/disease progression of rhabdomyosarcoma (RMS): A report from the Children’s Oncology Group (COG). J. Clin. Oncol..

[25] Mascarenhas L., Chi Y.Y., Hingorani P., Anderson J.R., Lyden E.R., Rodeberg D.A., Indelicato D.J., Kao S.C., Dasgupta R., Spunt S.L. (2019). Randomized Phase II Trial of Bevacizumab or Temsirolimus in Combination With Chemotherapy for First Relapse Rhabdomyosarcoma: A Report From the Children’s Oncology Group. J. Clin. Oncol. Off. J. Am. Soc. Clin. Oncol..

[26] Camero S., Ceccarelli S., De Felice F., Marampon F., Mannarino O., Camicia L., Vescarelli E., Pontecorvi P., Pizer B., Shukla R. (2019). PARP inhibitors affect growth, survival and radiation susceptibility of human alveolar and embryonal rhabdomyosarcoma cell lines. J. Cancer Res. Clin. Oncol..

[27] Tangutoori S., Baldwin P., Sridhar S. (2015). PARP inhibitors: A new era of targeted therapy. Maturitas.

[28] Murai J., Huang S.-Y.N., Das B.B., Renaud A., Zhang Y., Doroshow J.H., Ji J., Takeda S., Pommier Y. (2012). Trapping of PARP1 and PARP2 by Clinical PARP Inhibitors. Cancer Res..

[29] Norris R.E., Adamson P.C., Nguyen V.T., Fox E. (2014). Preclinical evaluation of the PARP inhibitor, olaparib, in combination with cytotoxic chemotherapy in pediatric solid tumors. Pediatr. Blood Cancer.

[30] Smith M.A., Hampton O.A., Reynolds C.P., Kang M.H., Maris J.M., Gorlick R., Kolb E.A., Lock R., Carol H., Keir S.T. (2015). Initial testing (stage 1) of the PARP inhibitor BMN 673 by the pediatric preclinical testing program: PALB2 mutation predicts exceptional in vivo response to BMN 673. Pediatr. Blood Cancer.

[31] Mangoni M., Sottili M., Salvatore G., Meattini I., Desideri I., Greto D., Loi M., Becherini C., Garlatti P., Delli Paoli C. (2018). Enhancement of Soft Tissue Sarcoma Cell Radiosensitivity by Poly(ADP-ribose) Polymerase-1 Inhibitors. Radiat. Res..

[32] Smith M.A., Reynolds C.P., Kang M.H., Kolb E.A., Gorlick R., Carol H., Lock R.B., Keir S.T., Maris J.M., Billups C.A. (2015). Synergistic Activity of PARP Inhibition by Talazoparib (BMN 673) with Temozolomide in Pediatric Cancer Models in the Pediatric Preclinical Testing Program. Clin. Cancer Res..

[33] Yan C., Brunson D.C., Tang Q., Do D., Iftimia N.A., Moore J.C., Hayes M.N., Welker A.M., Garcia E.G., Dubash T.D. (2019). Visualizing Engrafted Human Cancer and Therapy Responses in Immunodeficient Zebrafish. Cell.

[34] Farago A.F., Yeap B.Y., Stanzione M., Hung Y.P., Heist R.S., Marcoux J.P., Zhong J., Rangachari D., Barbie D.A., Phat S. (2019). Combination Olaparib and Temozolomide in Relapsed Small-Cell Lung Cancer. Cancer Discov..

[35] Kummar S., Chen A., Ji J., Zhang Y., Reid J.M., Ames M., Jia L., Weil M., Speranza G., Murgo A.J. (2011). Phase I study of PARP inhibitor ABT-888 in combination with topotecan in adults with refractory solid tumors and lymphomas. Cancer Res..

[36] Kummar S., Ji J., Morgan R., Lenz H.J., Puhalla S.L., Belani C.P., Gandara D.R., Allen D., Kiesel B., Beumer J.H. (2012). A phase I study of veliparib in combination with metronomic cyclophosphamide in adults with refractory solid tumors and lymphomas. Clin. Cancer Res. Off. J. Am. Assoc. Cancer Res..

[37] Middleton M.R., Friedlander P., Hamid O., Daud A., Plummer R., Falotico N., Chyla B., Jiang F., McKeegan E., Mostafa N.M. (2015). Randomized phase II study evaluating veliparib (ABT-888) with temozolomide in patients with metastatic melanoma. Ann. Oncol. Off. J. Eur. Soc. Med Oncol..

[38] Pietanza M.C., Waqar S.N., Krug L.M., Dowlati A., Hann C.L., Chiappori A., Owonikoko T.K., Woo K.M., Cardnell R.J., Fujimoto J. (2018). Randomized, Double-Blind, Phase II Study of Temozolomide in Combination With Either Veliparib or Placebo in Patients With Relapsed-Sensitive or Refractory Small-Cell Lung Cancer. J. Clin. Oncol. Off. J. Am. Soc. Clin. Oncol..

[39] Plummer R., Lorigan P., Steven N., Scott L., Middleton M.R., Wilson R.H., Mulligan E., Curtin N., Wang D., Dewji R. (2013). A phase II study of the potent PARP inhibitor, Rucaparib (PF-01367338, AG014699), with temozolomide in patients with metastatic melanoma demonstrating evidence of chemopotentiation. Cancer Chemother. Pharmacol..

[40] Su J.M., Thompson P., Adesina A., Li X.N., Kilburn L., Onar-Thomas A., Kocak M., Chyla B., McKeegan E., Warren K.E. (2014). A phase I trial of veliparib (ABT-888) and temozolomide in children with recurrent CNS tumors: A pediatric brain tumor consortium report. Neuro Oncol..

[41] Chugh R., Ballman K.V., Helman L.J., Patel S., Whelan J.S., Widemann B., Lu Y., Hawkins D.S., Mascarenhas L., Glod J.W. (2020). SARC025 arms 1 and 2: A phase 1 study of the poly(ADP-ribose) polymerase inhibitor niraparib with temozolomide or irinotecan in patients with advanced Ewing sarcoma. Cancer.

[42] Federico S.M., Pappo A.S., Sahr N., Sykes A., Campagne O., Stewart C.F., Clay M.R., Bahrami A., McCarville M.B., Kaste S.C. (2020). A phase I trial of talazoparib and irinotecan with and without temozolomide in children and young adults with recurrent or refractory solid malignancies. Eur. J. Cancer.

[43] Schafer E.S., Rau R.E., Berg S.L., Liu X., Minard C.G., Bishop A.J.R., Romero J.C., Hicks M.J., Nelson M.D., Voss S. (2020). Phase 1/2 trial of talazoparib in combination with temozolomide in children and adolescents with refractory/recurrent solid tumors including Ewing sarcoma: A Children’s Oncology Group Phase 1 Consortium study (ADVL1411). Pediatr. Blood Cancer.

[44] Chen X., Stewart E., Shelat A.A., Qu C., Bahrami A., Hatley M., Wu G., Bradley C., McEvoy J., Pappo A. (2013). Targeting oxidative stress in embryonal rhabdomyosarcoma. Cancer Cell.

[45] Shern J.F., Chen L., Chmielecki J., Wei J.S., Patidar R., Rosenberg M., Ambrogio L., Auclair D., Wang J., Song Y.K. (2014). Comprehensive Genomic Analysis of Rhabdomyosarcoma Reveals a Landscape of Alterations Affecting a Common Genetic Axis in Fusion-Positive and Fusion-Negative Tumors. Cancer Discov..

[46] Hettmer S., Liu J., Miller C.M., Lindsay M.C., Sparks C.A., Guertin D.A., Bronson R.T., Langenau D.M., Wagers A.J. (2011). Sarcomas induced in discrete subsets of prospectively isolated skeletal muscle cells. Proc. Natl. Acad. Sci. USA.

[47] Langenau D.M., Keefe M.D., Storer N.Y., Guyon J.R., Kutok J.L., Le X., Goessling W., Neuberg D.S., Kunkel L.M., Zon L.I. (2007). Effects of RAS on the genesis of embryonal rhabdomyosarcoma. Genes Dev..

[48] Le X., Pugach E.K., Hettmer S., Storer N.Y., Liu J., Wills A.A., DiBiase A., Chen E.Y., Ignatius M.S., Poss K.D. (2013). A novel chemical screening strategy in zebrafish identifies common pathways in embryogenesis and rhabdomyosarcoma development. Development.

[49] Li Z., Zhang Y., Ramanujan K., Ma Y., Kirsch D.G., Glass D.J. (2013). Oncogenic NRAS, required for pathogenesis of embryonic rhabdomyosarcoma, relies upon the HMGA2-IGF2BP2 pathway. Cancer Res..

[50] McKinnon T., Venier R., Dickson B.C., Kabaroff L., Alkema M., Chen L., Shern J.F., Yohe M.E., Khan J., Gladdy R.A. (2015). Kras activation in p53-deficient myoblasts results in high-grade sarcoma formation with impaired myogenic differentiation. Oncotarget.

[51] El-Badry O.M., Minniti C., Kohn E.C., Houghton P.J., Daughaday W.H., Helman L.J. (1990). Insulin-like growth factor II acts as an autocrine growth and motility factor in human rhabdomyosarcoma tumors. Cell Growth Differ. Mol. Biol. J. Am. Assoc. Cancer Res..

[52] Kalebic T., Tsokos M., Helman L.J. (1994). In vivo treatment with antibody against IGF-1 receptor suppresses growth of human rhabdomyosarcoma and down-regulates p34cdc2. Cancer Res..

[53] Maloney E.K., McLaughlin J.L., Dagdigian N.E., Garrett L.M., Connors K.M., Zhou X.M., Blättler W.A., Chittenden T., Singh R. (2003). An anti-insulin-like growth factor I receptor antibody that is a potent inhibitor of cancer cell proliferation. Cancer Res..

[54] Megiorni F., McDowell H.P., Camero S., Mannarino O., Ceccarelli S., Paiano M., Losty P.D., Pizer B., Shukla R., Pizzuti A. (2015). Crizotinib-induced antitumour activity in human alveolar rhabdomyosarcoma cells is not solely dependent on ALK and MET inhibition. J. Exp. Clin. Cancer Res..

[55] Scotlandi K., Manara M.C., Nicoletti G., Lollini P.L., Lukas S., Benini S., Croci S., Perdichizzi S., Zambelli D., Serra M. (2005). Antitumor activity of the insulin-like growth factor-I receptor kinase inhibitor NVP-AEW541 in musculoskeletal tumors. Cancer Res..

[56] Yohe M.E., Gryder B.E., Shern J.F., Song Y.K., Chou H.C., Sindiri S., Mendoza A., Patidar R., Zhang X., Guha R. (2018). MEK inhibition induces MYOG and remodels super-enhancers in RAS-driven rhabdomyosarcoma. Sci. Transl. Med..

[57] U.S. Food and Drug Administration FDA-Approved Drugs: Trametinib. https://www.accessdata.fda.gov/drugsatfda_docs/label/2013/204114s000lbl.pdf.

[58] U.S. Food and Drug Administration FDA-Approved Drugs: Selumetinib. https://www.accessdata.fda.gov/drugsatfda_docs/label/2020/213756s000lbl.pdf.

[59] U.S. Food and Drug Administration FDA-Approved Drugs: Binimetinib. https://www.accessdata.fda.gov/drugsatfda_docs/label/2019/210498s001lbl.pdf.

[60] U.S. Food and Drug Administration FDA-Approved Drugs: Cobimetinib. https://www.accessdata.fda.gov/drugsatfda_docs/label/2018/206192s002lbl.pdf.

[61] Banerjee A., Jakacki R.I., Onar-Thomas A., Wu S., Nicolaides T., Young Poussaint T., Fangusaro J., Phillips J., Perry A., Turner D. (2017). A phase I trial of the MEK inhibitor selumetinib (AZD6244) in pediatric patients with recurrent or refractory low-grade glioma: A Pediatric Brain Tumor Consortium (PBTC) study. Neuro Oncol..

[62] Dombi E., Baldwin A., Marcus L.J., Fisher M.J., Weiss B., Kim A., Whitcomb P., Martin S., Aschbacher-Smith L.E., Rizvi T.A. (2016). Activity of Selumetinib in Neurofibromatosis Type 1-Related Plexiform Neurofibromas. N. Engl. J. Med..

[63] Fangusaro J., Onar-Thomas A., Young Poussaint T., Wu S., Ligon A.H., Lindeman N., Banerjee A., Packer R.J., Kilburn L.B., Goldman S. (2019). Selumetinib in paediatric patients with BRAF-aberrant or neurofibromatosis type 1-associated recurrent, refractory, or progressive low-grade glioma: A multicentre, phase 2 trial. Lancet Oncol..

[64] Gross A.M., Wolters P.L., Dombi E., Baldwin A., Whitcomb P., Fisher M.J., Weiss B., Kim A., Bornhorst M., Shah A.C. (2020). Selumetinib in Children with Inoperable Plexiform Neurofibromas. N. Engl. J. Med..

[65] Perreault S., Larouche V., Tabori U., Hawkin C., Lippé S., Ellezam B., Décarie J.C., Théoret Y., Métras M., Sultan S. (2019). A phase 2 study of trametinib for patients with pediatric glioma or plexiform neurofibroma with refractory tumor and activation of the MAPK/ERK pathway: TRAM-01. BMC Cancer.

[66] Chen H.X., Sharon E. (2013). IGF-1R as an anti-cancer target—Trials and tribulations. Chin. J Cancer.

[67] Iams W.T., Lovly C.M. (2015). Molecular Pathways: Clinical Applications and Future Direction of Insulin-like Growth Factor-1 Receptor Pathway Blockade. Clin. Cancer Res. Off. J. Am. Assoc. Cancer Res..

[68] van Erp A.E.M., Versleijen-Jonkers Y.M.H., van der Graaf W.T.A., Fleuren E.D.G. (2018). Targeted Therapy–based Combination Treatment in Rhabdomyosarcoma. Mol. Cancer Ther..

[69] Pappo A.S., Vassal G., Crowley J.J., Bolejack V., Hogendoorn P.C., Chugh R., Ladanyi M., Grippo J.F., Dall G., Staddon A.P. (2014). A phase 2 trial of R1507, a monoclonal antibody to the insulin-like growth factor-1 receptor (IGF-1R), in patients with recurrent or refractory rhabdomyosarcoma, osteosarcoma, synovial sarcoma, and other soft tissue sarcomas: Results of a Sarcoma Alliance for Research Through Collaboration study. Cancer.

[70] Wan X., Yeung C., Heske C., Mendoza A., Helman L.J. (2015). IGF-1R Inhibition Activates a YES/SFK Bypass Resistance Pathway: Rational Basis for Co-Targeting IGF-1R and Yes/SFK Kinase in Rhabdomyosarcoma. Neoplasia.

[71] Ahmed S.R., Ball D.W., Cosgrove D., Scardina A., Petito E., Downs M., Nelkin B., Chen H.X., Doyle L.A., Donehower R.C. (2012). A phase I, single-institution, open-label, dose escalation trial with an expansion cohort evaluating the safety and tolerability of AZD6244 and IMC-A12 in subjects with advanced solid malignancies. J. Clin. Oncol..

[72] Knipstein J., Gore L. (2011). Entinostat for treatment of solid tumors and hematologic malignancies. Expert Opin. Investig. Drugs.

[73] Marampon F., Di Nisio V., Pietrantoni I., Petragnano F., Fasciani I., Scicchitano B.M., Ciccarelli C., Gravina G.L., Festuccia C., Del Fattore A. (2019). Pro-differentiating and radiosensitizing effects of inhibiting HDACs by PXD-101 (Belinostat) in in vitro and in vivo models of human rhabdomyosarcoma cell lines. Cancer Lett..

[74] Pham T.Q., Robinson K., Xu L., Skapek S.X., Chen E.Y. (2021). HDAC6 promotes self-renewal and migration/invasion of rhabdomyosarcoma. Oncogene.

[75] Tarnowski M., Tkacz M., Kopytko P., Bujak J., Piotrowska K., Pawlik A. (2019). Trichostatin A Inhibits Rhabdomyosarcoma Proliferation and Induces Differentiation through MyomiR Reactivation. Folia Biol..

[76] Abraham J., Nuñez-Álvarez Y., Hettmer S., Carrió E., Chen H.I., Nishijo K., Huang E.T., Prajapati S.I., Walker R.L., Davis S. (2014). Lineage of origin in rhabdomyosarcoma informs pharmacological response. Genes Dev..

[77] Gryder B.E., Wu L., Woldemichael G.M., Pomella S., Quinn T.R., Park P.M.C., Cleveland A., Stanton B.Z., Song Y., Rota R. (2019). Chemical genomics reveals histone deacetylases are required for core regulatory transcription. Nat. Commun..

[78] Bharathy N., Berlow N.E., Wang E., Abraham J., Settelmeyer T.P., Hooper J.E., Svalina M.N., Ishikawa Y., Zientek K., Bajwa Z. (2018). The HDAC3-SMARCA4-miR-27a axis promotes expression of the PAX3:FOXO1 fusion oncogene in rhabdomyosarcoma. Sci. Signal..

[79] Gryder B.E., Pomella S., Sayers C., Wu X.S., Song Y., Chiarella A.M., Bagchi S., Chou H.C., Sinniah R.S., Walton A. (2019). Histone hyperacetylation disrupts core gene regulatory architecture in rhabdomyosarcoma. Nat. Genet..

[80] Bharathy N., Berlow N.E., Wang E., Abraham J., Settelmeyer T.P., Hooper J.E., Svalina M.N., Bajwa Z., Goros M.W., Hernandez B.S. (2019). Preclinical rationale for entinostat in embryonal rhabdomyosarcoma. Skelet. Muscle.

[81] Kurmasheva R.T., Bandyopadhyay A., Favours E., Del Pozo V., Ghilu S., Phelps D.A., Erickson S.W., Peer C.J., Figg W.D., Smith M.A. (2019). Evaluation of entinostat alone and in combination with standard-of-care cytotoxic agents against rhabdomyosarcoma xenograft models. Pediatr. Blood Cancer.

[82] Tang F., Choy E., Tu C., Hornicek F., Duan Z. (2017). Therapeutic applications of histone deacetylase inhibitors in sarcoma. Cancer Treat. Rev..

[83] Yardley D.A., Ismail-Khan R.R., Melichar B., Lichinitser M., Munster P.N., Klein P.M., Cruickshank S., Miller K.D., Lee M.J., Trepel J.B. (2013). Randomized phase II, double-blind, placebo-controlled study of exemestane with or without entinostat in postmenopausal women with locally recurrent or metastatic estrogen receptor-positive breast cancer progressing on treatment with a nonsteroidal aromatase inhibitor. J. Clin. Oncol. Off. J. Am. Soc. Clin. Oncol..

[84] Fouladi M., Park J.R., Stewart C.F., Gilbertson R.J., Schaiquevich P., Sun J., Reid J.M., Ames M.M., Speights R., Ingle A.M. (2010). Pediatric phase I trial and pharmacokinetic study of vorinostat: A Children’s Oncology Group phase I consortium report. J. Clin. Oncol. Off. J. Am. Soc. Clin. Oncol..

[85] Malempati S., Chang B.H., Reid J.M., Liu X., Minard C.G., Keller C., Fox E., Weigel B. (2018). ADVL1513: Results of a phase 1 trial of entinostat, an oral histone deacetylase inhibitor, in pediatric patients with recurrent or refractory solid tumors. J. Clin. Oncol..

[86] Muscal J.A., Thompson P.A., Horton T.M., Ingle A.M., Ahern C.H., McGovern R.M., Reid J.M., Ames M.M., Espinoza-Delgado I., Weigel B.J. (2013). A phase I trial of vorinostat and bortezomib in children with refractory or recurrent solid tumors: A Children’s Oncology Group phase I consortium study (ADVL0916). Pediatr. Blood Cancer.

[87] Wood P.J., Strong R., McArthur G.A., Michael M., Algar E., Muscat A., Rigby L., Ferguson M., Ashley D.M. (2018). A phase I study of panobinostat in pediatric patients with refractory solid tumors, including CNS tumors. Cancer Chemother. Pharmacol..

[88] Zorzi A.P., Bernstein M., Samson Y., Wall D.A., Desai S., Nicksy D., Wainman N., Eisenhauer E., Baruchel S. (2013). A phase I study of histone deacetylase inhibitor, pracinostat (SB939), in pediatric patients with refractory solid tumors: IND203 a trial of the NCIC IND program/C17 pediatric phase I consortium. Pediatr. Blood Cancer.

[89] Lager J.J., Lyden E.R., Anderson J.R., Pappo A.S., Meyer W.H., Breitfeld P.P. (2006). Pooled analysis of phase II window studies in children with contemporary high-risk metastatic rhabdomyosarcoma: A report from the Soft Tissue Sarcoma Committee of the Children’s Oncology Group. J. Clin. Oncol. Off. J. Am. Soc. Clin. Oncol..

[90] Mascarenhas L., Lyden E.R., Breitfeld P.P., Walterhouse D.O., Donaldson S.S., Paidas C.N., Parham D.M., Anderson J.R., Meyer W.H., Hawkins D.S. (2010). Randomized phase II window trial of two schedules of irinotecan with vincristine in patients with first relapse or progression of rhabdomyosarcoma: A report from the Children’s Oncology Group. J. Clin. Oncol. Off. J. Am. Soc. Clin. Oncol..

[91] Santi D.V., Schneider E.L., Ashley G.W. (2014). Macromolecular Prodrug That Provides the Irinotecan (CPT-11) Active-Metabolite SN-38 with Ultralong Half-Life, Low Cmax, and Low Glucuronide Formation. J. Med. Chem..

[92] Ghilu S., Li Q., Fontaine S.D., Santi D.V., Kurmasheva R.T., Zheng S., Houghton P.J. (2020). Prospective use of the single-mouse experimental design for the evaluation of PLX038A. Cancer Chemother. Pharmacol..

[93] (2020). ProLynx Announces Phase 1B Clinical Trial of Its DNA-Damaging Agent PLX038 (PEG~SN-38) with the PARP Inhibitor Rubraca® (Rucaparib) at the National Cancer Institute.

[94] Moore A., Pinkerton R. (2009). Vincristine: Can its therapeutic index be enhanced?. Pediatric Blood Cancer.

[95] Horton T.M., Ames M.M., Reid J.M., Krailo M.D., Pendergrass T., Mosher R., Reaman G.H., Seibel N.L. (2008). A Phase 1 and pharmacokinetic clinical trial of paclitaxel for the treatment of refractory leukemia in children: A Children’s Oncology Group study. Pediatr. Blood Cancer.

[96] Jacobs S., Fox E., Krailo M., Hartley G., Navid F., Wexler L., Blaney S.M., Goodwin A., Goodspeed W., Balis F.M. (2010). Phase II trial of ixabepilone administered daily for five days in children and young adults with refractory solid tumors: A report from the children’s oncology group. Clin. Cancer Res. Off. J. Am. Assoc. Cancer Res..

[97] Bai R.L., Paull K.D., Herald C.L., Malspeis L., Pettit G.R., Hamel E. (1991). Halichondrin B and homohalichondrin B, marine natural products binding in the vinca domain of tubulin. Discovery of tubulin-based mechanism of action by analysis of differential cytotoxicity data. J. Biol. Chem..

[98] Towle M.J., Salvato K.A., Budrow J., Wels B.F., Kuznetsov G., Aalfs K.K., Welsh S., Zheng W., Seletsky B.M., Palme M.H. (2001). In vitro and in vivo anticancer activities of synthetic macrocyclic ketone analogues of halichondrin B. Cancer Res..

[99] Jordan M.A., Kamath K., Manna T., Okouneva T., Miller H.P., Davis C., Littlefield B.A., Wilson L. (2005). The primary antimitotic mechanism of action of the synthetic halichondrin E7389 is suppression of microtubule growth. Mol. Cancer Ther..

[100] Kolb E.A., Gorlick R., Reynolds C.P., Kang M.H., Carol H., Lock R., Keir S.T., Maris J.M., Billups C.A., Desjardins C. (2013). Initial testing (stage 1) of eribulin, a novel tubulin binding agent, by the pediatric preclinical testing program. Pediatr. Blood Cancer.

[101] Robles A.J., Kurmasheva R.T., Bandyopadhyay A., Phelps D.A., Erickson S.W., Lai Z., Kurmashev D., Chen Y., Smith M.A., Houghton P.J. (2020). Evaluation of Eribulin Combined with Irinotecan for Treatment of Pediatric Cancer Xenografts. Clin. Cancer Res..

[102] Schafer E.S., Rau R.E., Berg S., Liu X., Minard C.G., D’Adamo D., Scott R., Reyderman L., Martinez G., Devarajan S. (2018). A phase 1 study of eribulin mesylate (E7389), a novel microtubule-targeting chemotherapeutic agent, in children with refractory or recurrent solid tumors: A Children’s Oncology Group Phase 1 Consortium study (ADVL1314). Pediatr. Blood Cancer.

[103] Casanova M., Kramm C., Reinhardt D., Locatelli F., D’Adamo D.R., Scott R., Jia Y., Aluri J., Favre C., Bautista F. (2020). A phase I/II study of eribulin mesilate (ERI) plus irinotecan (IRI) in children with refractory or recurrent solid tumors. J. Clin. Oncol..

[104] Gill J., Zhang W., Zhang Z., Roth M., Harrison D.J., Rowshan S., Erickson S., Gatto G., Kurmasheva R., Houghton P. (2020). Dose-response effect of eribulin in preclinical models of osteosarcoma by the pediatric preclinical testing consortium. Pediatr. Blood Cancer.

[105] Isakoff M.S., Goldsby R., Villaluna D., Krailo M.D., Hingorani P., Collier A., Morris C.D., Kolb E.A., Doski J.J., Womer R.B. (2019). A phase II study of eribulin in recurrent or refractory osteosarcoma: A report from the Children’s Oncology Group. Pediatr. Blood Cancer.

[106] Cao L., Yu Y., Bilke S., Walker R.L., Mayeenuddin L.H., Azorsa D.O., Yang F., Pineda M., Helman L.J., Meltzer P.S. (2010). Genome-wide identification of PAX3-FKHR binding sites in rhabdomyosarcoma reveals candidate target genes important for development and cancer. Cancer Res..

[107] Crose L.E., Etheridge K.T., Chen C., Belyea B., Talbot L.J., Bentley R.C., Linardic C.M. (2012). FGFR4 blockade exerts distinct antitumorigenic effects in human embryonal versus alveolar rhabdomyosarcoma. Clin. Cancer Res. Off. J. Am. Assoc. Cancer Res..

[108] Khan J., Wei J.S., Ringnér M., Saal L.H., Ladanyi M., Westermann F., Berthold F., Schwab M., Antonescu C.R., Peterson C. (2001). Classification and diagnostic prediction of cancers using gene expression profiling and artificial neural networks. Nat. Med..

[109] deLapeyrière O., Ollendorff V., Planche J., Ott M.O., Pizette S., Coulier F., Birnbaum D. (1993). Expression of the Fgf6 gene is restricted to developing skeletal muscle in the mouse embryo. Development.

[110] Shaoul E., Reich-Slotky R., Berman B., Ron D. (1995). Fibroblast growth factor receptors display both common and distinct signaling pathways. Oncogene.

[111] Alijaj N., Moutel S., Gouveia Z.L., Gray M., Roveri M., Dzhumashev D., Weber F., Meier G., Luciani P., Rössler J.K. (2020). Novel FGFR4-Targeting Single-Domain Antibodies for Multiple Targeted Therapies against Rhabdomyosarcoma. Cancers.

[112] Cheuk A., Shivaprasad N., Schneider D., Yohe M., Tan M., Azorsa P., Sams R., Pomella S., Gryder B., Rota R. (2020). Abstract A08: Development of FGFR4-specific chimeric antibody receptor (CAR) T cell and bispecific T cell engager (BiTE) for rhabdomyosarcoma (RMS) immunotherapy. Cancer Res..

[113] Shivaprasad N., Xiong Y., Yohe M., Schneider D., Shern J., Baskar S., Dimitrov D., Sorenson P., Orentas R., Khan J. (2016). 649. Developing FGFR4 Chimeric Antigen Receptor CAR T Cell Therapy Against Rhabdomyosarcoma. Mol. Ther..

[114] Camero S., Camicia L., Marampon F., Ceccarelli S., Shukla R., Mannarino O., Pizer B., Schiavetti A., Pizzuti A., Tombolini V. (2020). BET inhibition therapy counteracts cancer cell survival, clonogenic potential and radioresistance mechanisms in rhabdomyosarcoma cells. Cancer Lett..

[115] Gryder B.E., Yohe M.E., Chou H.C., Zhang X., Marques J., Wachtel M., Schaefer B., Sen N., Song Y., Gualtieri A. (2017). PAX3-FOXO1 Establishes Myogenic Super Enhancers and Confers BET Bromodomain Vulnerability. Cancer Discov..

[116] Bid H.K., Phelps D.A., Xaio L., Guttridge D.C., Lin J., London C., Baker L.H., Mo X., Houghton P.J. (2016). The Bromodomain BET Inhibitor JQ1 Suppresses Tumor Angiogenesis in Models of Childhood Sarcoma. Mol. Cancer Ther..

[117] Sun Y., Han J., Wang Z., Li X., Sun Y., Hu Z. (2020). Safety and Efficacy of Bromodomain and Extra-Terminal Inhibitors for the Treatment of Hematological Malignancies and Solid Tumors: A Systematic Study of Clinical Trials. Front. Pharmacol..

[118] Pasquale E.B. (2010). Eph receptors and ephrins in cancer: Bidirectional signalling and beyond. Nat. Rev. Cancer.

[119] Berardi A.C., Marsilio S., Rofani C., Salvucci O., Altavista P., Perla F.M., Diomedi-Camassei F., Uccini S., Kokai G., Landuzzi L. (2008). Up-regulation of EphB and ephrin-B expression in rhabdomyosarcoma. Anticancer Res..

[120] Megiorni F., Gravina G.L., Camero S., Ceccarelli S., Del Fattore A., Desiderio V., Papaccio F., McDowell H.P., Shukla R., Pizzuti A. (2017). Pharmacological targeting of the ephrin receptor kinase signalling by GLPG1790 in vitro and in vivo reverts oncophenotype, induces myogenic differentiation and radiosensitizes embryonal rhabdomyosarcoma cells. J. Hematol. Oncol..

[121] Minami M., Koyama T., Wakayama Y., Fukuhara S., Mochizuki N. (2011). EphrinA/EphA signal facilitates insulin-like growth factor-I-induced myogenic differentiation through suppression of the Ras/extracellular signal-regulated kinase 1/2 cascade in myoblast cell lines. Mol. Biol. Cell.

[122] Randolph M.E., Cleary M.M., Bajwa Z., Svalina M.N., Young M.C., Mansoor A., Kaur P., Bult C.J., Goros M.W., Michalek J.E. (2017). EphB4/EphrinB2 therapeutics in Rhabdomyosarcoma. PLoS ONE.

[123] Saha N., Robev D., Mason E.O., Himanen J.P., Nikolov D.B. (2018). Therapeutic potential of targeting the Eph/ephrin signaling complex. Int. J. Biochem. Cell Biol..

[124] Xiao T., Xiao Y., Wang W., Tang Y.Y., Xiao Z., Su M. (2020). Targeting EphA2 in cancer. J. Hematol. Oncol..

